# Factors Associated with Beverage Intake in Low-Income, Overweight, or Obese Pregnant Women

**DOI:** 10.3390/nu14040840

**Published:** 2022-02-17

**Authors:** Mei-Wei Chang, Chyongchiou J. Lin, Rebecca E. Lee, Duane T. Wegener, Jie Hu, Karen Patricia Williams

**Affiliations:** 1 College of Nursing, The Ohio State University, 1585 Neil Avenue, Columbus, OH 43210, USA; lin.3782@osu.edu (C.J.L.); hu.1348@osu.edu (J.H.); williams.5963@osu.edu (K.P.W.); 2 Center for Health Promotion and Disease Prevention, Edson College of Nursing and Health Innovation, Arizona State University, 550 N. 3rd St., Phoenix, AZ 85004, USA; releephd@yahoo.com; 3 Department of Psychology, The Ohio State University, 1835 Neil Avenue, Columbus, OH 43210, USA; wegener.1@osu.edu

**Keywords:** sugar-sweetened beverages, poverty, stress, fruit juice

## Abstract

This study examined consumption proportions and factors associated with sugar-sweetened beverages (SSBs), artificially sweetened beverages (ASBs), and 100% fruit juice (FJ) consumption. We recruited Non-Hispanic Black (*n* = 136) and White (*n* = 192) low-income overweight or obese pregnant women aged 18 to 46 years (mean = 25.7 years) from the Special Supplemental Nutrition Program for Women, Infants, and Children clinics in Michigan, USA. Independent variables included weight status, trimester, smoking, stress, education, employment, race, and age. Dependent variables were high (consuming ≥ 1 serving/day) versus low consumptions of SSB, ASB, and 100% FJ. Multivariate logistic regression modeling was performed to examine factors associated with beverage consumption. Out of the sample, 48.2%, 6.7%, and 31.3% reported high SSB, ASB, and 100% FJ consumption, respectively. SSB consumption was associated with smoking (OR: 3.77, *p* < 0.001), education (OR: 0.57, *p* = 0.03), and race (OR: 1.69, *p* = 0.03). Artificially sweetened beverage consumption was not associated with any factors examined. One hundred percent FJ consumption was associated with stress (OR: 0.90, *p* = 0.03) and race (OR: 4.48, *p* < 0.001). Clinicians may advocate for reductions in SSB and 100% FJ consumption tailored to client consumption characteristics.

## 1. Introduction

Approximately 70% of American pregnant women consume higher than recommended amounts of added sugars (10% of total energy) [1]. The 2020–2025 Dietary Guidelines for Americans recommends that pregnant women limit intake of foods and beverages high in added sugars to prevent excessive gestational weight gain and promote favorable maternal and birth outcomes [1]. The largest dietary contributor of added sugar is sugar-sweetened beverages, SSBs, defined as any liquid with added sugars, for example, regular soda, sweetened juice beverages/drinks (fruit drinks), energy drinks, tea with added sugars, and coffee with added sugars [1].

Beverage intakes, such as sugar-sweetened beverages (SSBs), artificially sweetened beverages (ASBs), and 100% fruit juice (FJ), are modifiable factors affecting a pregnant women’s health [1]. High SSB consumption during pregnancy, defined as consuming at least 1 serving or 12 fl oz. (355 mL)/day [2,3], increases the risk for negative maternal and birth outcomes. High SSB consumption has been associated with poor dietary quality [3], increased caloric consumption [3], and excessive gestational weight gain [4]. Poor dietary quality and excessive gestational weight gain have been associated with being overweight or obese [5] and with increased risk for pre-eclampsia [5], gestational diabetes, and gestational hypertension [6]. High SSB consumption also increases a pregnant women’s risk of pre-eclampsia [7], the leading cause of maternal and perinatal morbidity and mortality and infant mortality [8]. Moreover, high SSB consumption during pregnancy has been associated with higher weight status in children (6 years old or younger) after adjusting for socio-demographic and lifestyle factors [9].

Similarly, high ASB consumption during pregnancy has been associated with increased risks of the infant being overweight [10], whereas 100% FJ is a good source of nutrients, as it contains minimal fiber. Moreover, high 100% FJ consumption has been associated with a significant increase in total daily caloric consumption [11], which is associated with excessive gestational weight gain. Due to the fact that high SSB, ASB, and 100% FJ consumption increases risks for negative maternal and child health outcomes, it is important to identify the proportion of three types of beverage consumption among pregnant women. However, limited information is available about beverage consumption among low-income overweight or obese pregnant women. A study of this population is important because they are at higher risks for gestational weight gain and negative maternal and birth outcomes compared to women with normal weight status [6,12]. Moreover, compared to their counterparts with incomes higher than 200% of the federally poverty level, low-income pregnant women were more likely to consume SSBs [3,13].

Currently, relatively few studies have examined factors associated with SSB, ASB, and 100% FJ consumption. Health status (such as weight status and trimester), individual behavior (such as smoking), and social factors (such as stress, education, employment, and race) might affect the three types of beverage consumption among pregnant women. Available data on pregnant women have shown associations between weight status and SSB consumption [13]. However, the associations between trimester and the three types of beverage consumption remain unknown. Prior studies of pregnant women have shown an association between smoking and SSB consumption [7,10] and between smoking and ASB consumption [7]. In terms of social factors, a previous focus group study of low-income overweight or obese postpartum reported that they tended to consume sweet foods/beverages when experiencing higher levels of stress [14]. However, the associations between stress and the three types of beverage consumption in pregnant women have not yet been investigated. Two studies of pregnant women have shown an association between education and SSB consumption [7,13]. However, the associations between education, ASB, and 100% FJ consumption and associations between employment and the three types of beverage consumption remain unknown. Only two studies of pregnant women examined the associations between race and SSB consumption and have reported mixed results [13,15].

Biology and genetics (such as age) might also influence the three types of beverage consumption. Currently, only two studies of pregnant women examined associations between age and SSB consumption and have yielded mixed results [13,15]. The associations between age, ASB, and 100% FJ consumption remain unknown.

This study investigated the proportion of SSB, ASB, and 100% FJ consumption among low-income overweight or obese pregnant women. We also applied four categories of health determinants [16] (health status, individual behavior, social factors, and biology and genetics) to explore whether these categories would be associated with high SSB, ASB, and 100% FJ consumption. We hypothesize that health status (higher weight status and earlier trimester), individual behavior (current smoking), social factors (higher stress score, lower education, unemployment, and Black race), and biology and genetics (younger age) would associate with high SSB, ASB, and 100% FJ consumption.

## 2. Methods

### 2.1. Study Design, Participants, and Recruitment

This cross-sectional study utilized convenience sampling. Participants were recruited from 4 WIC (the Special Supplemental Nutrition Program for Women, Infants, and Children) clinics located in western and southern Michigan. WIC provides a nutrition education and food voucher/package to pregnant, postpartum, and lactating women and children under 5 years old from low-income environments. Detailed descriptions of study recruitment have been previously published [17]. Briefly, the trained recruiters personally invited pregnant women waiting for their WIC appointment to participate in the study in 2011. In order to be eligible to participate, women had to be pregnant, Non-Hispanic Black or White (hereafter, Black or White), at least 18 years old, and have a pre-pregnancy weight status (body mass index, BMI) of at least 25.0 kg/m^2^. Qualified women provided a written consent form prior to participation followed by completing a self-administered pencil-and-paper survey while waiting for their WIC appointment. This study was conducted according to the guidelines laid down in the Declaration of Helsinki, and all procedures involving research study participants were approved by the Institutional Review Board at Michigan State University (IRB #10-761, 03/30/11).

### 2.2. Measures

Demographics and BMI. Participants self-reported the following: smoking (never smoked, smoked and quit, and current smoking), education (less than high school, high school graduate, some college education, and at least college education), and employment (e.g., employed part-and full-time, homemakers, and unemployed). Participants also reported race/ethnicity, age, and last menstrual cycle, which was used to calculate trimester status. Moreover, participants self-reported height and weight, both of which were used to calculate BMI.

Perceived Stress (Hereafter, Stress)**.** The Perceived Stress Scale (9 items) with reported validity (validated using number of life events: *r* = 0.17–0.39) and reliability (Cronbach Alpha = 0.84–0.86) [18] was used to measure stress. The survey has been used to collect data from low-income pregnant women [19]. Participants were asked about their feelings and thoughts during the last month [18]. Responses were rated on a 4-point scale: 1 = rarely or never to 4 = usually or always. The overall stress score was the sum of the 9 items. Higher scores represented higher levels of stress.

SSB, ASB, and 100% FJ Consumption. A beverage intake survey with acceptable validity and reliability (*R*^2^ = 0.52–0.95, *p* < 0.001), validated using 24-hour dietary recalls, was used to measure beverage consumption [20]. The survey includes 15 beverage items (e.g., water, vegetable juice, 100% fruit juice, milk, and soda). Participants reported their frequency and amount of beverage consumption in the past month. Responses to the frequency of each beverage consumed were rated on a 7-point scale: 1 = never or less than 1 time per week; 2 = 1 time per week; 3 = 2–3 times per week; 4 = 4–6 times per week; 5 = 1 time per day; 6 = 2 times per day; and 7 = at least 3 times per day. Responses to the amount of each beverage consumed each time were rated on a 5-point scale: 1 = less than 6 fl oz. (177 mL, 3/4 cup); 2 = 8 fl oz. (237 mL, 1 cup); 3 = 12 fl oz. (355 mL, 1 ½ cups); 4 =16 fl oz. (473 mL, 2 cups); and 5 = at least 20 fl oz. (591 mL, 2 ½ cups). If the participants never drank a specific beverage in the past month (the frequency response), they skipped the amount response, which was coded as 0. In order to compute beverage consumption, frequency was multiplied by amount with higher scores indicating higher consumption [20]. Consistent with prior studies [3,7], the present study included 8 items: SSBs (6 items: regular soda, fruit drinks, tea beverages with sugars, coffee beverage with added sugars, energy drinks, and meal replacement shakes/protein drinks), ASB (1 item: diet soda), and 100% FJ (1 item).

### 2.3. Statistical Analysis

A total of 332 pregnant women responded to the survey, but the analysis included 328 women. Four women (1.2%) did not fill out any beverage intake survey questions because they had to leave. Of the data collected, only the beverage intake survey had missing data (<0.1%). Hot Deck imputation was applied to impute missing data. Statistical software SAS version 9.4 (SAS Institute, Inc. 2013, Cary, NC, USA) was used for all analytical procedures. Descriptive analysis was performed to examine demographics and the proportion of the 3 types of beverage consumption. One serving of beverage intake, defined as 12 fl oz, was used as a cut-off value [2,3] to dichotomize low (<1 serving per day = 0) and high (≥1 serving per day = 1) consumptions. Multivariate logistic regression modeling was conducted to explore factors associated with daily SSB, ASB, and 100% FJ consumption, all of which were dependent variables. Independent variables included weight status (obesity versus overweight), trimester (second or third trimester versus the first trimester), smoking (current smoking versus non-smoking (never smoked, smoked and quit smoking)), stress (total score), education (at least some college education versus high school or less education), employment (employed versus unemployed and homemakers, hereafter unemployed), race (Black versus White), age (<25 years old versus ≥25 years old). Pearson correlations were used to explore associations among SSB, ASB, and 100% FJ consumption (treated as continuous variables). Statistical significance was set at *p* < 0.05 for all tests.

## 3. Results

### 3.1. Demographics and Proportion of SSB, ASB, and 100% FJ Consumption

Table 1 summarizes demographics and stress scores of the study participants. Most participants were at least 25 years old; White; had at least some college education; were unemployed; non-smoking; and were in high weight status (BMI ≥ 30 kg/m^2^ prior to pregnancy). More than one-third of participants were in the second trimester. Table 2 presents proportion of the three types of beverage consumption. Of the study sample, 48.2% reported high SSB consumption, 6.7% reported high ASB consumption, and 31.3% reported high 100% FJ consumption. Most SSB consumption was from regular soda followed by fruit drinks and tea with added sugars. Results showed significant associations between SSB and ASB consumption (*r* = 0.27, *p* < 0.001), between SSB and 100% FJ consumption (r = 0.43, *p* < 0.001), and between ASB and 100% FJ consumptions (*r* = 0.17, *p* = 0.002).

### 3.2. Factors Associated with Beverage Consumptions

Table 3 presents odds ratios (ORs) and 95% confidence intervals (CIs) based on multivariate logistic regressions in which all independent variables were simultaneously used to predict high consumption of each type of beverage. Health status (weight status and trimester) was not significantly associated with high consumption of the three types of beverages. Individual behavior (current smoking, OR: 3.77; 95% CI: 1.80–7.66, *p* < 0.001) was associated with high SSB consumption. In terms of social factors, higher levels of stress (OR: 0.90; 95% CI: 0.83–0.99, *p =* 0.03) were associated with low 100% FJ consumption, and at least some college education (OR: 0.57; 95% CI: 0.35–0.94, *p* = 0.03) was associated with low SSB consumption. However, being Black was associated with high SSB (OR: 1.69; 95% CI: 1.04–2.74, *p* = 0.03) and 100% FJ consumption (OR: 4.48; 95% CI: 2.61–7.69, *p* < 0.001). Biology and genetics were not associated with high consumption of any of the three types of beverages.

## 4. Discussion

This study examined consumption proportion and factors associated with daily SSB, ASB, and 100% FJ consumption among low-income, overweight, or obese pregnant women. Our study findings partially supported our hypotheses: current smoking, lower education, and Black race were associated with higher SSB consumption. In addition, Black race was associated with higher consumptions of 100% FJ.

In the 2009 WIC food package, policy makers reduced the 100% FJ allotment to align with the Dietary Guidelines for Americans [21]. Currently, each pregnant woman enrolled in WIC receives up to 144 fl oz. (4259 mL) of 100% FJ per month or 4.8 fl oz. (142 mL)/day [22]. In 2017, the National Academies of Science, Engineering, and Medicine recommended further revision of the WIC food package, for example, increasing fruit and vegetable intake as a trade-off for 100% FJ, to make the WIC food package even more consistent with the Dietary Guidelines for Americans.

Our results showed that almost half of the sample reported high SSB consumption, which is about 2.5-times higher than a previous study of pregnant women that included all weight status and income levels [23]. The present study found that a small proportion of the study sample reported high ASB consumption and nearly one-third of pregnant women reported high 100% FJ consumption. It is possible that these women perceived the benefits of drinking 100% fruit juice for fetus growth and development. However, they might not be aware of the importance of portion control. Moreover, they might have used 100% fruit juice to substitute for whole fruits because of the perception that juice is relatively cheaper and easier to consume than whole fruits. Moreover, they might have misinterpreted or not fully understood dietary messages related to fruit consumption and portion size [24]. We also found that high SSB consumption was moderately associated with high 100% FJ consumption. Our findings support a need to help low-income overweight or obese pregnant women in reducing SSB and 100% FJ consumption. However, a recent systematic review showed that reducing beverage consumption has been overlooked by most prior dietary intervention studies for healthy pregnancy [25].

We found no significant associations between weight status and any of the three types of beverage consumption, a finding that contradicts findings of a previous study of pregnant women across all income levels [13]. The inconsistency might have related to differences in demographics and analyses. Whereas the present study treated weight status as a categorical variable (overweight versus obesity) and only included women with a pre-pregnancy BMI of at least 25.0 kg/m^2^, the previous study included all weight statuses and treated weight status as a continuous variable. Thus, associations between weight status and beverage consumption in previous work might have reflected the larger range of weight status included in the study. We found that current smokers were more likely than nonsmokers to report high SSB consumption, which is consistent with prior studies [10,13]. However, smoking was not related to ASB or 100% FJ consumption in the present study.

The study findings revealed that stress was only associated with 100% FJ consumption but not with consumption of the other 2 types of beverages. Comparison of our stress findings to previous studies is not feasible because we are unaware of other studies of pregnant women that investigated such associations. Our findings revealed that women with at least some college education were less likely to report high SSB consumption than women with high school or less education. This finding is consistent with a prior study of pregnant women [13], but it is contradictory to another study [15]. Moreover, we found that Black women were 1.6-times more likely than White women to report high SSB consumption, which is consistent with one prior study of pregnant women [13] but inconsistent with another study of pregnant women [15]. Our finding might be related to the area of residence and low-income status of most of the Black women in the present study (dense, urban city). Living in dense, urban cities can increase one’s exposure and access to fast food restaurants and convenience stores [26,27], which has been associated with more SSB consumption [28]. A prior study on neighborhood socioeconomic position on dietary intake among African Americans in the Jackson Heart Study (N = 3948 with 64% Black women) found that Blacks residing in a neighborhood with lower socioeconomic position was associated with higher SSB intake [29]. Moreover, we found that race did not play a role in ASB consumption, yet Black women were almost 4.5-times more likely than White women to report high 100% FJ consumption. Factors contributing to these disparities remain unknown. Black women suffer a disproportionally high prevalence of obesity [30], which increases their risk for excessive gestational weight gain and its associated adverse maternal and birth outcomes [5,6]. Our findings highlight health disparities and point to the importance of tailored interventions for low-income, overweight, or obese Black pregnant women to reduce 100% FJ consumption. Prior studies of pregnant women that examined the association between age and SSB consumption have also yielded mixed results [13,15].

The present study has several limitations. The cross-sectional design precludes any cause–effect conclusions. The secondary nature of the analysis means that the study was not originally designed with tests of independent variables of beverage consumption in mind. Our study is limited in the number of individual behavior or social factors that might influence the study outcomes. The selection of possible factors was limited by the original design purposes (that were not primarily to address beverage consumption patterns). Data were collected via self-report, all of which are subject to potential inaccuracy of dietary recall. Low-income women and individuals with higher weight status are more likely to under-report their dietary intake [31]. The data were also collected in 2011. Thus, the present data might only provide an approximate understanding of the proportion and amount of each type of beverage consumption and provide a baseline of consumption against which current and future data might be compared. This study used self-reported height and weight, which might have resulted in misclassification of some women into different weight categories. Finally, we only included non-Hispanic Black and White pregnant women because our collaborative sites served a very small proportion of pregnant women with other racial/ethnic backgrounds.

The present study has some strengths. We collected frequency and the amount of beverage consumption. Thus, we were able to report daily consumption amount. We also used reliable and valid surveys to measure perceived stress and beverage consumption. The study included a homogenous but vulnerable sample: low-income overweight or obese pregnant women.

## 5. Implications for Research and Practice

Based on our findings, future prospective studies are needed in order to examine the associations between high SSB and 100% FJ consumption, dietary quality, and gestational weight gain among low-income, overweight, or obese pregnant women. Moreover, focus group discussions and future prospective studies might consider identifying factors (e.g., physical and social environment and process of decision making) influencing Black pregnant women’s SSB and 100% FJ consumption. Moreover, lifestyle intervention studies promoting healthy pregnancies among low-income pregnant women might consider including reductions in SSB and 100% FJ consumption. In 2018, WIC served 675,227 pregnant women nationwide [32]. Registered dietitians and registered dietitian nutritionists working at WIC as well as clinicians working with the study population at prenatal care clinics might consider discussing SSB and 100% FJ consumption with their clients, especially those who are smokers or are Black. Perhaps providing tailored education to reduce consumption could be beneficial. The present study findings, including a moderate association between SSB and 100% FJ consumption, might also bring policy makers’ and nutrition educators’ attention to providing practical and realistic strategies to reduce 100% FJ and SSB consumption beyond removing 100% FJ from the WIC food package.

## Figures and Tables

**Table 1:** Descriptive characteristics of low-income, overweight, or obese pregnant women (N = 328).

Continuous Variables	Mean (SD)	Range
Total stress score ^1^	20.4 (3.0)	13–30
Weight status defined as body mass index (kg/m^2^)	32.5 (6.2)	25.0–60.3
Age (years)	25.7 (5.6)	18–46
Gestational weeks	19.4 (9.9)	3–39
Categorical Variables	*N*	%
Smoker		
Non-smoker	280	85.4
Current smoker	48	14.6
Education		
≤High school	129	39.3
≥Some college	199	60.7
Employment Status		
Unemployed	212	64.6
Employed	116	35.4
Race		
Non-Hispanic White	192	58.5
Non-Hispanic Black	136	41.5
Age		
<25 years old	163	49.7
≥25 years old	165	50.3
Trimester Status		
First trimester: ≤12 weeks	106	32.3
Second trimester: 13–27 weeks	129	39.3
Third trimester: ≥28 weeks	93	28.4

^1^ Perceived Stress Scale was used [18]. Characteristics were self-reported by the participants. Descriptive analysis was performed.

**Table 2:** Beverage consumption of low-income, overweight, or obese Pregnant Women (N = 328).

Type of Beverage	Serving/Day ^1^					
	Mean (SD)	Range	0	0.01–0.99	1.00–1.99	≥2.00
			*N* (%)	*N* (%)	*N* (%)	*N* (%)
Sugar-sweetened beverages	1.7 (2.3)	0–17	26 (7.9)	144 (43.9)	74 (22.6)	84 (25.6)
Artificially sweetened beverages	0.2 (0.6)	0–5	160 (48.8)	146 (44.5)	7 (2.1)	15 (4.6)
100% fruit juice	0.9 (1.1)	0–5	15 (4.6)	210 (64.0)	45 (13.7)	58 (17.7)

^1^ One serving/day is defined as 12 fl oz. (355 mL)/day. Sugar-sweetened beverages include regular soda (M = 0.7 (SD = 1.1); fruit drinks (0.6 (1.1)), tea with added sugars (0.3 (0.8)), coffee with added sugars (0.1 (0.4)), energy drinks (0.0 (0.0)), and meal replacement shakes/protein drinks (0.0 (0.0)). Beverage intakes were self-reported by participants. Descriptive analysis was performed.

**Table 3:** Estimates of logistic regressions by group of beverage intake (*N* = 328).

Independent Variables	Outcome Variables					
	Sugar-Sweetened Beverage		Artificial Sugar Sweetened Beverage		100% Fruit Juice	
	Odds Ratio (95% CI)	*p* Value	Odds Ratio (95% CI)	*p* Value	Odds Ratio (95% CI)	*p* Value
Health						
BMI category (ref: overweight)						
Obesity	0.87 (0.55–1.39)	0.5637	1.75 (0.68–4.51)	0.2434	1.21 (0.72–2.02)	0.4727
Trimester (ref: ≤12 weeks)						
13–27 weeks	1.60 (0.92–2.79)	0.0930	1.00 (0.34–2.96)	0.9997	1.25 (0.68–2.30)	0.4657
≥28 weeks	1.20 (0.66–2.18)	0.5442	1.32 (0.43–4.05)	0.6230	0.82 (0.42–1.61)	0.5644
Individual behaviors						
Smoking (ref: non-smoker)						
Current smoker	3.77 (1.86–7.66)	0.0002	1.06 (0.32–3.47)	0.9242	1.19 (0.56–2.52)	0.6580
Social factors						
Total stress score	0.97 (0.90–1.05)	0.4918	1.06 (0.91–1.23)	0.4777	0.90 (0.83–0.99)	0.0262
Education (ref: ≤ high school)						
Some college and higher	0.57 (0.35–0.94)	0.0272	0.56 (0.22–1.41)	0.2195	0.62 (0.36–1.06)	0.0782
Employment (ref: unemployed)						
Employed	1.26 (0.78–2.06)	0.3443	0.70 (0.26–1.91)	0.4885	1.07 (0.63–1.83)	0.8045
Race						
Black Race (ref: White)	1.69 (1.04–2.74)	0.0339	0.69 (0.26–1.86)	0.4652	4.48 (2.61–7.69)	<0.0001
Biology						
Age (ref: ≥25 years)						
Under 25 years old	1.00 (0.62–1.60)	0.9975	0.52 (0.20–1.34)	0.1776	0.80 (0.47–1.34)	0.3958

## Data Availability

Not applicable.
